# The Emerging Role of Epigenetics in Therapeutic Targeting of Cardiomyopathies

**DOI:** 10.3390/ijms22168721

**Published:** 2021-08-13

**Authors:** Christina Pagiatakis, Vittoria Di Mauro

**Affiliations:** 1 IRCCS-Humanitas Research Hospital, Via Manzoni 56, 20089 Rozzano, Italy; 2 Institute of Genetic and Biomedical Research (IRGB), Milan Unit, National Research Council, Via Fantoli 16/15, 20138 Milan, Italy

**Keywords:** cardiomyopathies, epigenetics, non coding RNAs (ncRNAs), omics approaches

## Abstract

Cardiomyopathies (CMPs) are a heterogeneous group of myocardial diseases accountable for the majority of cases of heart failure (HF) and/or sudden cardiac death (SCD) worldwide. With the recent advances in genomics, the original classification of CMPs on the basis of morphological and functional criteria (dilated (DCM), hypertrophic (HCM), restrictive (RCM), and arrhythmogenic ventricular cardiomyopathy (AVC)) was further refined into genetic (inherited or familial) and acquired (non-inherited or secondary) forms. Despite substantial progress in the identification of novel CMP-associated genetic variations, as well as improved clinical recognition diagnoses, the functional consequences of these mutations and the exact details of the signaling pathways leading to hypertrophy, dilation, and/or contractile impairment remain elusive. To date, global research has mainly focused on the genetic factors underlying CMP pathogenesis. However, growing evidence shows that alterations in molecular mediators associated with the diagnosis of CMPs are not always correlated with genetic mutations, suggesting that additional mechanisms, such as epigenetics, may play a role in the onset or progression of CMPs. This review summarizes published findings of inherited CMPs with a specific focus on the potential role of epigenetic mechanisms in regulating these cardiac disorders.

## 1. Introduction

Cardiomyopathies (CMPs) are a group of heterogeneous genetic or idiopathic cardiac dysfunctions that include structural and functional myocardial abnormalities related to myocyte injuries [1]. The anatomical changes and cell death responsible for the defective electrical contraction of the heart often correlate with arrhythmias and consequent heart failure [2]. Historically, the World Health Organization (WHO) mainly defined CMPs on the basis of dominant pathophysiology and etiological/pathogenic factors, with an initial classification into hypertrophic, dilated, and restrictive categories [3]. However, recent advantages in omics approaches significantly improved the knowledge of molecular mechanisms underlying both genetic and acquired cardiomyopathies, prompting the American Heart Association (AHA) to refine the classification system in 2006 [2]. CMPs are now categorized as primary or secondary. In primary CMPs, the disease process is confined to the heart, while secondary CMPs encompass a larger class of conditions in which cardiac involvement can occur as generalized, systemic, or even multiorgan dysfunction. In particular, primary CMPs can be further classified into (i) genetic, (ii) acquired, or (iii) mixed in etiology. Genetic CMPs are related to mutations that affect the heart, while, in the acquired phenotype, the nongenetic causes lead to cardiac complications. In 2007, the European Society of Cardiology (ESC) proposed a novel classification in which each morpho-functional subtype was further classified on the basis of familial/genetic and nonfamilial/nongenetic forms. The familial/genetic forms were divided into unidentified gene defects and specific disease subtypes, while the nonfamilial/nongenetic forms were divided into idiopathic and specific disease subtypes [4].

**Figure 1:**
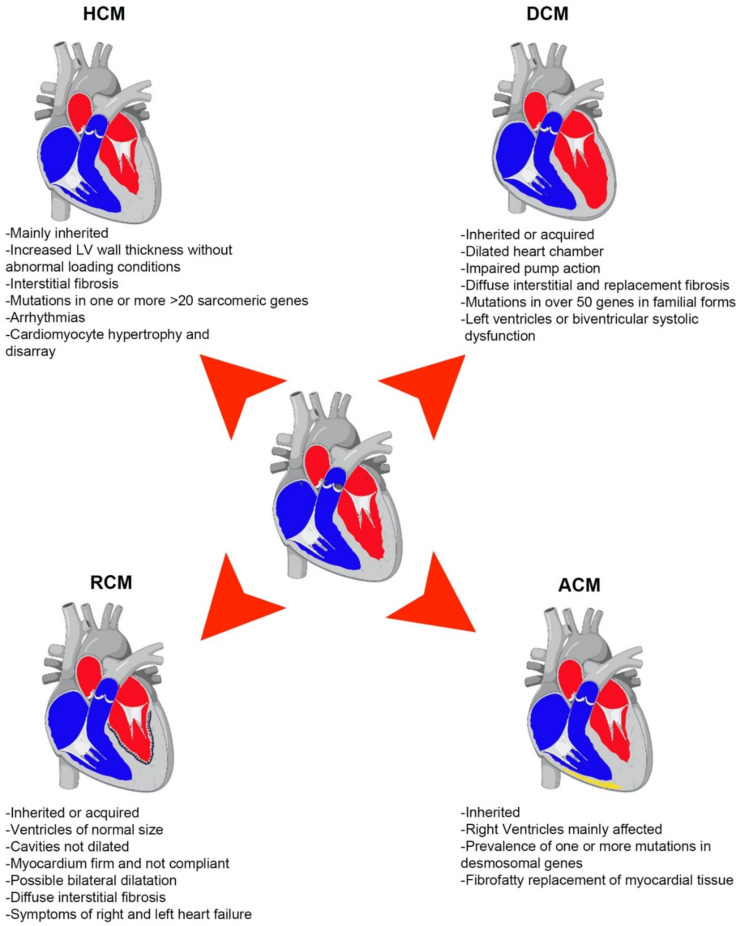
Schematic representation of major types of cardiomyopathies (CMPs). Common characteristics and phenotypes of DCM, HCM, RCM, and ACM. Heart images were created using biorender.com (https://biorender.com/; accessed on 11 August 2021).

Irrespective of the heterogeneity in phenotypes and etiologies, the most common CMPs, including hypertrophic, dilated, and restrictive CMPs, often share signs and symptoms that ultimately lead to heart failure, thus pointing out the possibility of shared dysregulation in key molecular mechanisms that control heart function (Figure 1).

### 1.1. Types of Primary CMPs

#### 1.1.1. Dilated Cardiomyopathy (DCM)

Dilated cardiomyopathy (DCM) represents the predominant type of CMPs worldwide, affecting approximately 1/2500 people, with a higher frequency of diagnosis in adult males between the ages of 20 and 50 years [2]. It is characterized by an increase in both myocardial mass and volume, with thin and stretched walls, abnormal loading conditions, and/or coronary artery disease; such phenotypes ultimately result in left-ventricular (LV) dysfunction [5]. DCM is also one of the leading causes of heart failure and the most common reason for heart transplantation [6]. Currently, two-dimensional echocardiography is the gold standard for DCM diagnosis; diagnostic criteria rely on a left-ventricular ejection fraction (LVEF) less than 45%, with a left-ventricular end-diastolic dimension greater than 112% of that predicted for age and body surface area [7]. Genetically inherited mutations in genes encoding cytoskeletal (*ACTC1*, actin alpha cardiac muscle 1), sarcomere (*TTN*, titin; *MYH6*, myosin 6; *TNNT2*, troponin T), calcium (Ca^2+^) handling (*PLN*, cardiac phospolamban), and nuclear envelope (*LMNA*, Prelamin A/C) proteins, to name a few, account for up to 35% of cases [8]. However, many other factors, including myocarditis, exposure to alcohol, drugs, and toxins, and metabolic and endocrine disturbances are also responsible for the onset of DCM [5]. The 5 year survival rate after a diagnosis of DCM is approximately 50%, as patients often develop progressive congestive heart failure (CHF) and other complications including thromboembolic conditions and arrhythmia [9]. 

#### 1.1.2. Hypertrophic Cardiomyopathy (HCM)

Hypertrophic cardiomyopathy (HCM) is the most common genetic form of inherited heart disease, with a prevalence of 1 in 500, and it is responsible for sudden death in people <50 years of age, especially young athletes [2]. More recent epidemiological studies reported that approximately 20 million people worldwide are affected by HCM, although many people suffering from HCM remain undiagnosed, with 10% of cases clinically identified and 6% symptomatic [10].

HCM is associated with mutations in one or more of the genes encoding proteins of the cardiac sarcomere, Z-disc, and calcium-controlling proteins. Mutations of cardiac sarcomere protein genes with an autosomal dominant pattern of inheritance are responsible for up to 60% of HCM in adults. The most common mutations are related to genes encoding the heavy chains of β-myosin (*MYH7*) and myosin-binding protein C (*MYBPC3*), while genes related to cardiac troponin I and T (*TNNI3*, *TNNT2*), tropomyosin α-1 chain (*TPM1*), and myosin light chain 3 (*MYL3*) are less commonly involved [11,12]. The presence of sarcomere protein mutations is associated with more severe hypertrophy, myocardial fibrosis, and increased prevalence of sudden cardiac death (SCD) [13]. Other genetic disorders including Fabry’s disease, amyloidosis, Danone’s disease, and Friederich’s ataxia may be related to HCM (5–10% of patients) [11]. Ultimately 25–30% of HCM cases have an unknown etiology (sporadic cases). 

#### 1.1.3. Restrictive Cardiomyopathy (RCM)

Restrictive cardiomyopathy (RCM) is the least common form of heart muscle disease in adults in the developed world, with controversy surrounding its proper definition, epidemiology, and diagnostic criteria [14]. Indeed, the exact prevalence and incidence of RCM in the general population is still undefined because of the lack of large population-based epidemiology studies.

To date, only a few studies have estimated that, in Western countries, RCM accounts for less than 5% of cardiomyopathic disorders and approximately 2.5–5% of all pediatric CMPs [15]. The prognosis of RCM-affected people is very poor, with a mortality rate of 50% within 2 years of diagnosis [16]. 

RCMs can be classified into primary and secondary. The primary form of RCM (e.g., endomyocardial fibrosis, Löffler’s endocarditis, idiopathic restrictive cardiomyopathy) is defined by the presence of hemodynamic abnormalities in the absence of histological features that indicate myocardial abnormalities, while secondary RCMs are often a result of other cardiac or systemic disorders (e.g., amyloidosis, sarcoidosis, and Fabry’s disease) adversely affecting the filling pattern of the left ventricle [17]. Several mutations were found in the sarcomeric subunits of troponin T (*TNNT2*), troponin I (*TNNI3*), α-actin (*ACTC*), and β-myosin heavy chain (*MYH7*) [18].

So far, no effective treatment other than heart transplantation is available for RCM-affected patients, who generally die from congestive heart failure or ischemia-related sudden death [2].

#### 1.1.4. Arrhythmogenic Cardiomyopathy (ACM)

Arrhythmogenic cardiomyopathy (ACM) is defined as an arrhythmogenic disorder of the myocardium not secondary to ischemic, hypertensive, or valvular heart disease [19]. The population incidence of ACM has been estimated at 1:1000 to 5000 worldwide; it is considered one of the leading causes of sudden cardiac death (SCD) in people aged ≤35 years, and it is responsible for up to 10% of deaths of undiagnosed cardiac disease in the <65 age group [20,21].

Approximately half of ACM cases are considered to be familial, most frequently inherited in an autosomal-dominant fashion with variable penetrance, although autosomal-recessive patterns have also been reported [22]. Up to 60% of ACM cases harbor one or more mutations in genes encoding for major components of the cardiac desmosomes, such as plakoglobin (*JUP*), desmoplakin (*DSP*), plakophilin-2 (*PKP2*), desmoglein-2 (*DSG2*), and desmocollin-2 (*DSC2*) [22]. Additional mutations in genes encoding non-desmosomal proteins, such as phospholamban (*PLN*), the cardiac ryanodine receptor (*RYR2*), transforming growth factor (*TGF-β3*), transmembrane protein-43 (*TMEM43*), and the cardiac sodium channel (*SCN5A*), have also been associated with ACM [23]. Given these mutations, the hallmark of this disease is represented by a progressive, focal replacement of cardiomyocytes with poorly conductive adipose and fibrotic (fibrofatty) tissue that impairs electrical conduction, leading to tachycardia, myocyte loss, and atrophy of the ventricular myocardium [2]. 

Due to the lack of a unique diagnostic tool, the current strategy for a proper ACM diagnosis relies on a combination of different sources of clinical information, including genetic, electrocardiographic, arrhythmic, morpho-functional, and histopathological findings [24]. The main goal of treatment is the prevention of SCD. Current therapeutic strategies include lifestyle changes, β-blockers, antiarrhythmic drugs (AADs), and catheter ablation. However, an implantable cardioverter defibrillator is so far the only proven lifesaving therapy, despite the fact that it is also correlated with high morbidity because of device-related post-surgery complications or inappropriate implantable cardioverter defibrillator interventions [20,24]. Therefore, elucidating the underlying molecular mechanisms of CMPs could provide novel therapeutic strategies for the treatment of these diseases (Table 1).

### 1.2. Epigenetic Mechanisms

Epigenetics are mechanisms regulating gene expression independently of changes to DNA sequence, which can be grouped into four main processes: DNA methylation, histone modifications, chromatin remodeling, and noncoding RNAs. These mechanisms modulate chromatin structure and regulate transcription factor binding in order to regulate gene expression. Moreover, these mechanisms serve for both immediate/short-term and acute changes to gene expression. Acute changes in the epigenome and their upstream signals have been associated with physiological and pathological stimuli, as well as to environmental factors such as diet, stress, physical activity, smoking, and alcohol consumption [25,26,27]. Furthermore, perturbations to such mechanisms have been directly linked with changes in cellular and organ function upon pathological onset.

#### 1.2.1. DNA Methylation

DNA modifications are covalent modifications of DNA bases whereby the attachment of a methyl group to the 5-carbon in cytosine-paired-with-guanine (CpG) dinucleotide sequences occurs [28]. CpG islands are found in the majority of promoters in both human and mouse genomes. Methylation of cytosines has been largely associated with transcriptional repression, as they act as docking sites of methyl-CpG-binding domain (MBD) proteins, which promote the formation of silent chromatin. In mammals, DNA methylation patterns are established and maintained by three DNA methyltransferase enzymes (DNMTs); DNMT3A and DNMT3B are essential for de novo DNA methylation during development [29], while DNMT1 is required for maintaining methylation patterns during cell division. Another important mechanism is that of DNA hydroxymethylation, which is the product of hydroxylation of 5-mC (5-methylcytosine) catalyzed by Ten-eleven translocation (TET) enzymes. High levels of 5-hmC (5-hydroxymethylcytosine) in promoter and enhancer regions are generally linked with high levels of transcription [30]. Global changes in DNA methylation have been correlated with the onset of several cardiovascular pathologies [31].

#### 1.2.2. Histone Modifications

Histone modifications are covalent post-translational modifications to histone proteins, including acetylation, methylation, phosphorylation, ubiquitylation, and sumoylation [25,32]. Histone acetylation occurs on lysine residues of histone tails, allowing transcription factor accessibility, and it is a dynamic process which depends on the activity of two classes of enzymes, histone acetyltransferases (HATs) and histone deacetylases (HDACs) [25]. Histone methylation is another important epigenetic mechanism, which affects transcriptional levels depending on the position and degree of lysine and arginine methylation on histone tails. Histone methylation associated with transcription activation occurs as di- or trimethylation of histone H3 at lysines 4, 36, and 79 (H3K4, H3K36, and H3K79, respectively) and monomethylated H3K9 and H4K20, whereas transcriptional repression is characterized by trimethylation of H3K9, H3K27, and H4K20, and dimethylation of H3K9. These modifications result in gene silencing through formation of facultative and constitutive heterochromatic regions [33]. Histone methylation is also a dynamic process, resulting from the activity of two classes of enzymes: histone methyltransferases (HMTs) and histone demethylases (HDMs) [25]. HATs and HDACs have been highly implicated in pathophysiological onset. The p300 histone acetyltransferase has been shown to function as a transcriptional coactivator for several transcription factors by bridging DNA-binding transcription factors to basal transcriptional machinery, in addition to serving as a scaffold for other transcription factors. 

#### 1.2.3. Chromatin Remodeling

The transcriptional state of gene expression is highly maintained through the conformation of chromatin structure. This is important in the strict regulation of the transcriptional state of genes in a cell- and tissue-specific manner, and it is catalyzed, in part, by ATP-dependent chromatin-remodeling complexes. These complexes are formed by several proteins that regulate gene expression by modifying the nucleosome organization of DNA, using energy derived from ATP hydrolysis. Transcriptional activators function by promoting the formation of an open and accessible chromatin structure, allowing the recruitment of transcription factors involved in positive regulation of transcription. One such family of proteins is the SWI/SNF (Switch/Sucrose Nonfermentable) complex, which promotes the formation of open-conformation chromatin. Conversely, other chromatin remodeling factors cause gene silencing by organizing the nucleosomes on DNA in a highly compact form, thus preventing accessibility of transcription factors [34]. The SWI/SNF complex, in parallel with BRG1 (Brahma-related gene 1), has been widely described in cardiac development and cardiovascular hypertrophy and disease [35,36]. Interestingly, a recent study showed that BRG1 maintains cardiomyocyte homeostasis by regulating cardiomyocyte mitophagy and mitochondrial dynamics [37]. 

#### 1.2.4. Noncoding RNAs

Another very prominent class of epigenetic mechanisms is represented by noncoding RNAs (ncRNAs), which comprise the majority of our genome and are not translated into proteins, accounting for a myriad of regulatory mechanisms and pathways at the level of the epigenome. Noncoding RNAs are generally classified on the basis of their length (short and long). The class of short ncRNAs includes molecules of RNA shorter than 200 nucleotides such as PIWI-interacting RNAs, small interfering RNAs (siRNAs), and microRNAs (miRNAs). On the other hand, long ncRNAs (lncRNAs) include molecules of RNA longer than 200 nucleotides, which are poorly conserved at the DNA sequence level but are promising targets for epigenetic and genetic regulation. Long ncRNAs can regulate the expression of proteins at the transcriptional and translational levels. MicroRNAs are mainly known to inhibit the expression of genes by binding to the 3′-UTR (untranslated region) of their target mRNAs, resulting in degradation of the target mRNA and subsequent inhibition of protein translation. Their role in regulating target genes at the transcriptional level has also been reported [27,38]. Several ncRNAs have been implicated in the regulation of key pathways and enzymes involved in several cardiomyopathies, as discussed below more in detail.

**Figure 2:**
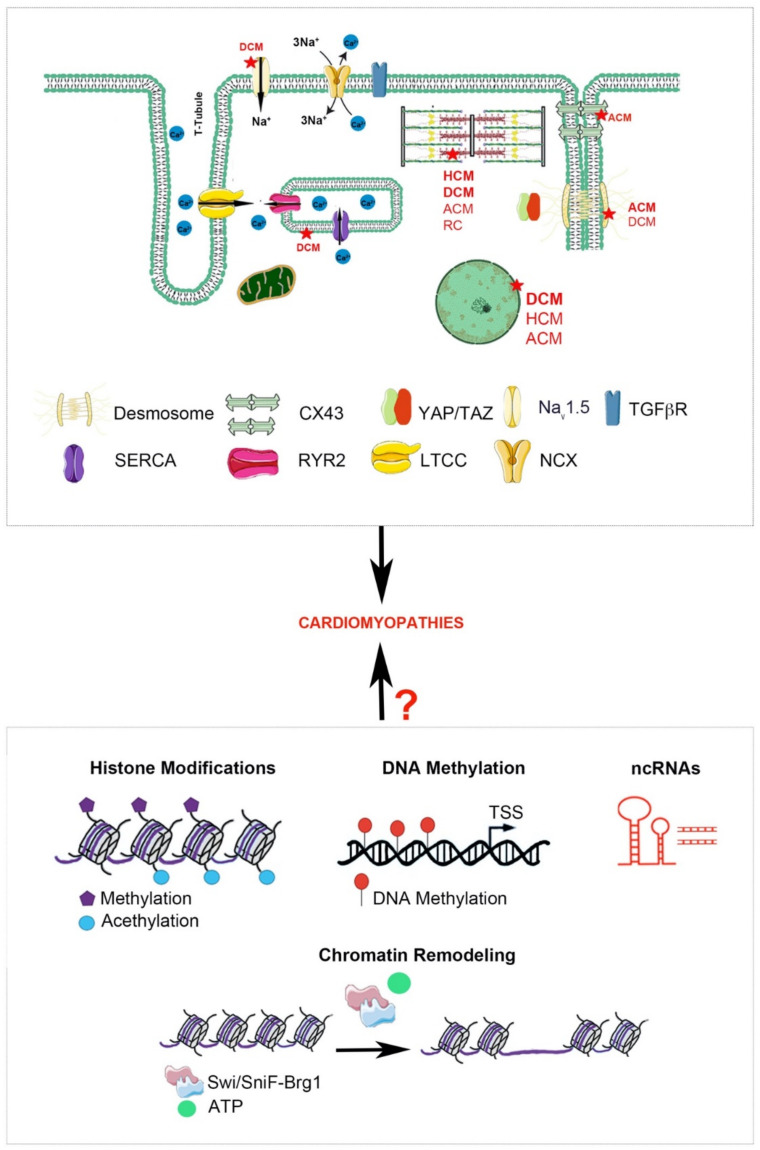
Schematic depiction of the multiple potential mechanisms implicated in the pathophysiology of CMPs. Most representative genetic mutations found in all types of CMPs (Top). Representation of potential epigenetic mechanisms regulating the onset/progression of CMPs (Bottom). This figure was created using https://smart.servier.com/ (accessed on 4 July 2021).

Therefore, the purpose of this review was to corroborate the molecular and genetic mediators currently known to underlie the onset and development of primary CMPs, by exploring potential epigenetic pathways involved in the molecular control of pathogenesis (Figure 2).

## 2. Molecular and Epigenetic Mechanisms in CMPs

### 2.1. Dilated Cardiomyopathy

#### 2.1.1. Molecular Mechanisms in DCM: An Overview

DCM can be characterized by left-ventricular dilation, which is associated with systolic dysfunction, further leading to diastolic dysfunction and impaired right-ventricular function. The major forms of DCM are inherited, and advanced genome-wide association studies have found more than 40 genes implicated in the development and pathophysiology of this disease. Most mutations have been correlated with genes encoding cytoskeletal and sarcomeric proteins. Truncation mutations in the titin gene (*TTN*) have been associated with over 27% of DCM cases [39], although mutations in noncontractile proteins, such as the cochaperone for heat-shock protein 70 (*HSP70*) and heat-shock cognate 70 chaperone proteins (encoded by *BAG3*), have also been reported [8]. Furthermore, alterations in nuclear envelope proteins (*LNMA A/C*), the calcium cycling gene phospholamban (*PLN*), and the RNA splicing gene *RBM20* have also been reported [40]. The clinical outcome of these mutations is a reduced force transmission and/or resistance to mechanical stress of cardiomyocytes, as well as altered electrical conduction, leading to defects in cell signaling pathways that modify cardiac function [41]. From a molecular point of view, changes in the structural architecture of the cardiomyocyte lead to a global remodeling of the myocardium, which further results in molecular perturbations causative of necrotic and fibrotic patch formation and calcification, which contribute to the cardiac dysfunction associated with DCM.

At the molecular level, the underlying mechanisms still remain largely unknown; however, several signaling pathways have been associated with this pathogenesis. For example, TGFβ (transforming growth factor β) is highly implicated in the activation of fibroblasts and the fibrotic response; both TGFβ and IL-11 (interleukin 11), which have been shown to activate TGFβ, are induced in the diseased heart. This activation has been associated with fibrosis formation and myocardial remodeling through regulation of extracellular matrix proteins [42]. 

Despite the fact that most cases of DCM have been shown to be a result of genetic mutations in several sarcomeric genes, the molecular perturbations associated with these mutations are now slowly emerging, although most remain elusive. Moreover, very little is known about the epigenetic mechanisms that contribute to the pathogenesis of DCM, including fibrosis, inflammation, and metabolic changes; such biomolecular mediators regulating gene expression will be of critical importance in the development of potential pharmacological therapies. 

#### 2.1.2. DNA Methylation in DCM

DNA methylation is critical in the regulation of gene expression, and its aberrant regulation has been associated with pathogenesis in various contexts. Genome-wide methylation studies have shown that levels of DNA methylation were globally altered in end-stage cardiomyopathic hearts, specifically at CpG islands [43]. A recent study by Meder et al. used a multi-omics study in DCM patients, identifying epigenetic susceptibility regions and novel biomarkers linked to myocardial dysfunction and heart failure. In this study, 59 DCM-associated epigenetic loci were determined to be significantly altered in DCM patients, an effect which was observed not only from myocardial tissue, but also from peripheral blood, indicating that such DNA methylation patterns could be potential biomarkers as diagnostic tools of DCM [44]. The specific role of DNA methyltransferases (DNMTs) in the onset and/or regulation of DCM has not yet been described. One study describing a fatal dilated cardiomyopathy, Keshan disease (KD), outlined the role of selenium deficiency as a main cause in this pathophysiology. Using MeDIP-ChIP (methylated DNA immunoprecipitation (MeDIP) chromatin immunoprecipitation), this study revealed altered DNA methylation patterns in the promoter regions of *TLR2* (Toll-like receptor 2) and *ICAM1* (intercellular adhesion molecule 1) genes, an effect that was mediated by DNMT1, revealing a potential role of DNA methylation during the inflammatory response in the myocardium [45]. The TET family of enzymes catalyzes the deposition of 5-methylcytosine (5mC) and 5-hydroxymethylcytosine (5hmC), regulating global DNA methylation patterns. A recent study combining RNA- and ChIP-sequencing revealed altered 5mC and 5hmC levels in DCM hearts. The genes that were identified in this analysis were correlated with inflammation, tissue fibrosis, cell death, cardiac remodeling, and cardiomyocyte growth and differentiation. Such phenotypes are correlated with DCM, and this study identified the epigenetic regulation of intronic regions via altered DNA methylation patterns of pathways involved in cardiac remodeling and contractile dysfunction [46]. Therefore, it will be of great interest to further study the role of the Ten-eleven translocation (TET) family of enzymes in DCM pathogenesis, as well as their role in altering molecular pathways involved in this disease. 

Although the regulation of DNA methylation remains largely unknown in DCM, such studies underline the importance of uncovering the epigenetic mechanisms involved in order to identify potential therapeutic targets.

#### 2.1.3. Histone Modifications in DCM

Histone modifications regulate gene expression primarily through the activity of histone acetyltransferases (HATs), histone deacetylases (HDACs), and histone methyl transferases (HMTs) by post-translationally modifying histone tails. In a study of end-stage nonischemic DCM patients, global changes in histone modifications were observed. This study showed an overall reduction in H3 lysine 4 trimethylation (H3K4me3), H3 lysine 9 dimethylation (H3K9me2), and H3 lysine 9 trimethylation (H3K9me3) [47], outlining a correlation between global changes in histone modifications and the onset of DCM. A key epigenetic enzyme that has previously been implicated in the control of cardiomyocyte homeostasis is the histone methyltransferase G9a (Ehmt2, euchromatic histone lysine methyltransferase 2), through the deposition of H3K9me2. Such repressive marks are necessary to maintain heterochromatic regions (gene silencing) of the fetal gene program that is reactivated during pressure overload [33]. Interestingly, G9a is significantly decreased in a rat DCM model, resulting in increased expression of cell adhesion molecules, which is an important pathway involved in the onset of DCM [48]. Another histone methyltransferase which has been implicated in DCM is DOT1L, which catalyzes the methylation of H3 at lysine 79 (H3K79me); expression of DOT1L is decreased in DCM hearts, and a murine knockout model of DOT1L has been associated with a DCM phenotype [49]. Although the specific mechanisms and regulation of gene expression of these enzymes and other histone methyltransferases in DCM remain elusive, it will be interesting to further investigate the role of these factors as biomolecular mediators of DCM.

The roles of HDACs and HATs have been widely described in cardiovascular diseases as “writers” and “erasers” of histone acetylation, in the control of the epigenetic landscape of the heart. Very little is known about the specific mechanisms of these enzymes in the pathogenesis of DCM. Murine models of HDAC1 and HDAC2 knockouts presented with dilated cardiomyopathy phenotypes, resulting from, among other factors, an upregulation of gene expression related to contractile proteins and calcium channels [50]. A recent study showed that the use of the HDAC inhibitor suberoylanilide hydroxamic acid (SAHA) promoted cardiomyogenic differentiation; in the context of DCM, this could be a very promising therapeutic target for activating the regenerative potential of hmMSCs (myocardium-derived mesenchymal stem cells) in the dilated myocardium [51]. One study outlined a potential role of the histone acetyltransferase EP300 in the regulation of the myosin binding protein C cardiac isoform (*MYBPC3*), which is a thick filament accessory protein of the striated muscle sarcomere A band. Mutations in *MYBPC3* result in contractile defects in DCM. This study used an in vitro model knockout model of MYBPC3, MYBPC3^−/−^, in hiPSC-CMs (human induced pluripotent stem-cell-derived cardiomyocytes), in order to evaluate its role in contractile deficits in the myocardium. The increase in EP300 expression in MYBPC3^−/−^ cardiac microtissues suggested a potential epigenetic mechanism via which mechanical overload in the MYBPC3 deficient tissue, leading to contractile deficits, could be a result of aberrant histone acetylation [52]. Although the role of EP300, in concomitance with Gata4, has been elucidated in cardiac hypertrophy [53], its role in cardiomyopathies remains highly elusive. 

Therefore, the involvement of histone acetylation in DCM has proven to be a potentially interesting avenue as a potential biomolecular mediator of cardiomyopathies. Using such enzymes in mouse models of DCM could provide us with a great amount of information for their use as therapeutic targets (Table 2). 

#### 2.1.4. Chromatin Remodeling in DCM

Although the mechanisms of chromatin remodeling have been widely studied in cardiac development, little is known regarding the role of this epigenetic mechanism in DCM. One of the few studies regarding the cardiac specific Brg1/Baf60C complex showed that the depletion of Baf60c (mammalian chromatin remodeling complex BRG1-associated factor 60C) in cardiomyocytes results in postnatal dilated cardiomyopathy [34] in a mechanism involving myocardin, a cardiac- and smooth muscle-specific transcription factor involved in cardiac development and homeostasis [62]. Changes in chromatin structure and accessibility could be critical in linking genetic mutations to global epigenetic changes in DCM. 

#### 2.1.5. Noncoding RNAs in DCM

The role of noncoding RNAs as epigenetic biomolecules has been gaining importance over the last several years, not only as regulators of transcription, but also as potential biomarkers for disease. In the context of DCM, the dysregulation of several miRNAs has been associated with the onset of pathogenesis; however, a plethora of studies have mainly explored the relationship between miRNA profiles in DCM patients without a detailed definition of the etiological mechanisms. A genome-wide profiling of miRNAs in human LV identified miR-17-5p, miR-28, and miR-106a as differentially expressed in DCM samples compared to control samples [63]. Moreover, the cardiac-specific miR-208 has been shown to be upregulated in biopsy tissues from DCM patients, having a negative association with clinical outcome [64]. Amongst miRNAs with known cardiac-enriched expression, miRNA-1 and the miR-19 family were downregulated in DCM, whereas miR-241 was found to be upregulated [65]. Several clinical cohorts have exhibited various circulating miRNAs associated with DCM, some of which include miR-21, miR-26, miR-29, miR-30, and miR-133a [66], to name a few; however, little is known about the molecular mechanism through which these and other miRNAs contribute to pathogenesis. It will be necessary to elucidate these mechanisms using high-throughput omics approaches in order to further delineate their role in this heterogeneous disease.

Long noncoding RNAs (lncRNAs) are also crucial epigenetic regulators and, unlike miRNAs, are an extremely heterogeneous subset of noncoding RNAs that exert their function in a myriad of mechanisms, including epigenetic regulation of transcription, RNA processing, and translation [27]. Although lncRNAs have been gaining great interest in cardiovascular disease over the last several years, very little is known about their role in cardiomyopathies. An important lncRNA, *H19*, which was amongst the first lncRNAs described, is highly conserved, and it has been highly implicated in embryonic development and growth. In the heart, it has also been shown to be involved in cardiac endothelial aging, mineralization of aortic valves, and ischemia/reperfusion-evoked apoptosis. Moreover, polymorphisms of the *H19* gene have been reported to alter levels of *H19*, which are associated with risk factors for cardiovascular diseases, such as high blood pressure [67]. Interestingly, *H19* was significantly upregulated in the myocardium of a rat model of DCM, promoting cardiomyocyte apoptosis, although the molecular mechanism remains to be fully elucidated [68]. Another recent study showed that the lncRNA *HAND2-AS1* may participate in end-stage dilated cardiomyopathy by interacting indirectly with IGF-1 (insulin-like growth factor 1). Plasma levels of IGF-1 and *HAND2-AS1* were significantly lower in end-stage DCM patients than in healthy controls and were positively correlated in end-stage DCM patients. A follow-up study revealed that low levels of IGF-1 or *HAND2-AS1* were significantly associated with poor survival [69]. Although little is known about lncRNAs in DCM, a recent study used genome-wide profiling of circulating lncRNAs in DCM by RNA-sequencing and explored the potential functions of these lncRNAs in DCM using bioinformatic analysis [70]. These findings, therefore, provide a critical stepping stone for determining the role of these lncRNAs as important biomolecular epigenetic mediators of DCM onset.

### 2.2. Hypertrophic Cardiomyopathy

#### 2.2.1. Molecular Mechanisms in HCM: An Overview

The cellular and molecular mechanisms associated with this cardiomyopathy range from hypertrophy to fetal gene program activation, metabolic perturbations, fibrosis, contractile dysfunction, and impaired calcium handling. One important mutation associated with severe forms of hypertrophy is beta myosin heavy chain (β-MHC) or *MYH7*, which is important in the regulation of the actin–myosin interaction and functional contractility of the cardiomyocyte. Mutations in *MYH7* results in ventricular tachycardia, high risk of sudden death, earlier disease onset, and greater penetrance [71]. Another key mutation determining HCM pathophysiology is that of the cardiac myosin-binding protein C (*MYBPC3*), which accounts for 60–70% of identified HCM cases. 

In addition to the known causal genetic mutations resulting in structural and functional changes of sarcomeric proteins, various molecular pathways have been implicated in the onset of HCM, although molecular and epigenetic perturbations in this pathology remain highly elusive. An important phenotype observed in HCM patients is the elevation of inflammatory cytokines and infiltration of leucocytes into the myocardium. These molecular events have been associated with the progression of this pathophysiology, via Fas (sFas), Fas ligand (sFas-L), TNFα (tumor necrosis factor α), and IL-6 (interleukin-6), which are negatively correlated with fractional shortening and result in heart failure [72]. Studies on endomyocardial samples from HCM patients showed myocyte hypertrophy, myofiber disarray, fibrosis, and inflammatory cell infiltration, resulting from nuclear factor kappa B (NF-κB) and inflammatory interleukin activation. As expected, this study showed that the inflammatory response in these patient samples was associated with myocardial fibrosis [73]. This fibrotic phenotype in response to inflammatory cytokines has been shown to be a predictor of ventricular arrhythmias, potentially resulting from a dysregulated sarcomere, tissue injury, and mitochondrial stress [72]. Therefore, these mechanisms need to be further investigated in order to elucidate their epigenetic regulation as potential targets for reversing HCM phenotypes.

#### 2.2.2. DNA Methylation in HCM

The role of DNA methylation remains widely unstudied in the context of HCM; however, it is important to investigate such processes in order to further elucidate the pathways involved in this pathophysiology. One study outlined the role of DNA methyltransferase 1 (Dnmt1) in murine HF models; this study showed that Dnmt1 is upregulated in rat heart failure and cardiac injury models, correlating with an upregulation of DNMT1 in samples from HCM patients. Using a myocardium-specific knockout model of Dnmt1 in rats, this study showed that absence of Dnmt1 results in resistance to cardiac pathological stress and prevents gene reprogramming associated with HF by activating pathways involved in myocardial protection and anti-apoptosis. Using genome-wide transcriptomic analysis, it was shown that Dnmt1 knockout regulates global DNA methylation [74]. 

Therefore, it is necessary to further investigate the role of DNA methylation in the propagation of the HCM phenotype, specifically in response to stress and cardiac injury, in order to further elucidate the epigenetic mechanisms involved in the pathogenesis of HCM. 

#### 2.2.3. Histone Modifications in HCM

The role of histone modifications in the development of HCM pathology has yet to be elucidated. Using knowledge of regulation of gene expression by histone acetylases and deacetylates in the context of cardiovascular disease will be a key starting point for further understanding of these mechanisms in the context of HCM.

One study revealed the role of the histone acetyltransferase p300 as a key player in Duchenne muscular dystrophy, in which the main cause of death is cardiomyopathy. This study revealed that the NAD^+^-dependent protein deacetylase SIRT1 (Sirtuin 1) induces p300 downregulation via a ubiquitin/proteasome-dependent mechanism. The findings of this study implicate the role of histone acetylation, specifically through the upregulation of p300, in the diseased heart. [54]. The role of p300 and histone acetylation described in this context will be a critical direction for further investigation in HCM patients.

Histone deacetylases have been shown to play a key role in cardiac homeostasis and hypertrophy. Class IIa HDACS play a key role in cardiomyocyte hypertrophy; inhibiting their activity augments the hypertrophic response. For example, inhibition of HDAC2 induces cardiac hypertrophy, while its activation plays a critical role in the regulation of antihypertrophic gene expression [75]. The role of HDACs has not been explicitly studied in HCM; since the mechanisms of action of these epigenetic enzymes are widely known in the context of hypertrophy, using HCM patient samples to determine the expression of class II HDACs and, conversely, HATs (such as p300) will provide further insight into the epigenetic mechanisms involved in HCM. Furthermore, since HDAC inhibitors are currently being used in studies for the treatment of various pathologies, a clearer elucidation of these pathways is necessary in the context of HCM (Table 2). 

#### 2.2.4. Chromatin Remodeling in HCM

The role of chromatin remodeling in HCM has not been clearly elucidated, and this epigenetic mechanism requires further study in the context of this disease. Studies have shown that BRG1 is activated in certain patients with hypertrophic cardiomyopathy and promotes embryonic-like expression patterns in cardiomyocytes, such as a shift between α-MHC and β-MHC isoforms. In the embryo, Brg1is necessary for promoting myocyte proliferation via activation of Bmp10 (bone morphogenetic protein 10) and suppression of p57. Moreover, it interacts with HDACs and PARP (poly(ADP-ribose) polymerase) to repress αMHC and activate βMHC in order to promote fetal cardiomyocyte differentiation. In the adult heart, BRG1 is not active, and its activation in HCM is associated with α-MHC and β-MHC switching, indicating that aberrant chromatin remodeling mechanisms are involved in cardiomyopathic pathophysiology. This study suggests that targeting Brg1 could be a favorable mechanism in controlling the switch between α-MHC and β-MHC, thus reducing the HCM phenotype [76].

Epigenetic mechanisms are dynamic processes, in which combined changes at different levels of modifications regulate gene expression. As a bridging point between histone modifications and chromatin structure, one recent study described a mechanism via which stress activates the expression of Brg1, G9a/Glp (histone methyltransferase), and Dnmt3 (DNA methyltransferase) in the cardiomyocyte. Results from this study revealed that activation of Brg1 recruits G9a and then Dnmt3, resulting in deposition of H3K9 and CpG methylation, respectively. This results in silencing of Myh6 and impairment of cardiac contraction. This study could be key for further elucidating this mechanism in hypertrophic cardiomyopathy [55].

#### 2.2.5. Noncoding RNAs in HCM

Noncoding RNAs have been widely shown to be key epigenetic players in the control of many pathophysiologies, and their involvement in cardiomyopathies has slowly emerged in the last several years. miR-126, which has been shown to be essential for development by activating the survival kinases ERK1/2 (mitogen-activated protein kinases) and Akt (protein kinase B), is involved in a cardioprotective phenotype by activating cell survival and proangiogenic pathways during ischemia, through the modulation of HATs and HDACs [77], providing another potential link between histone modifications and noncoding RNA in the regulation of the HCM phenotype. 

A very recent study by Gao et al. revealed miRNA expression profiles in HCM patients. This study found that miR-487a, miR-654, miR-30d, miR-154, miR-3193, and miR-3671 were significantly modulated in HCM patients. These miRNAs have also been studied in the context of acute heart failure and myocardial fibrosis, to name a few. These miRNAs were correlated with the cardiovascular system and modulation of cardiac function. Moreover, they were implicated in the β-catenin signaling pathway, voltage-gated calcium channel gene regulation, and fibrosis [78]. Another recent study performed a meta-analysis of published databases in order to delineate miRNAs associated with hypertrophic cardiomyopathy. Their dataset selection, extraction, and analysis, combining 68 different studies and 329 patients, found 87 differentially expressed miRNAs in HCM patients. The most commonly reported miRNAs in these datasets were mir-21, mir-29a, and mir-133. Several of the miRNAs, including mir-1-3p, mir-19b, mir-21, mir-29a, mir-155, and mir-221, were related to either hypertrophy or fibrosis. Mir-29a showed a more consistent phenotypic correlation with HCM and could be a potential biomarker [79] (Table 3).

Long noncoding RNAs are now emerging as key epigenetic regulators of a myriad of pathophysiologies; in the same study mentioned above by Gao et al., lncRNA expression profiles of HCM patients were revealed. A total of 1482 protein-coding genes were found nearby differentially expressed lncRNAs. Several of these lncRNAs were found to be located in the introns or nearby genes related to several CMPs, including *TTN*, *ACTC1*, *TPM1*, *JPH2*, *ANKRD1*, *DTNA*, and *FHL2* [78]. Further investigation of these lncRNAs will be critical to further uncover the molecular and epigenetic mechanisms contributing to HCM, specifically with respect to fibrotic and inflammatory phenotypes. The lncRNA *MIAT*, was found to regulate fibrosis in hypertrophic cardiomyopathy in patients presenting with a fibrotic phenotype. This research showed that miR-29a and *MIAT* were differentially expressed between the fibrosis HCM group and the nonfibrotic control HCM group, indicating that *MIAT* could promote the development of fibrosis by negatively regulating the expression of miR-29a [82]. The role of the lncRNA *H19,* also implicated in DCM, was found to be implicated in HCM, whereby single-nucleotide polymorphisms in *H19* were determined to have a significant association with risk of developing HCM. Further molecular studies need to be conducted in order to determine the pathways that are dysregulated in patients with these mutations [83].

These initial studies on noncoding RNAs in the context of HCM are extremely promising steps for further elucidating molecular and epigenetic perturbations of the pathophysiology outside of the causative genetic mutations.

### 2.3. Restrictive Cardiomyopathy

#### 2.3.1. Molecular Mechanisms in RCM: An Overview

Restrictive cardiomyopathy is the least frequent amongst the cardiomyopathies, and little is known about its genetic, molecular, and epigenetic etiology. A few studies have started to outline some key insights with respect to molecular mechanisms involved in RCM; however, further research into the mutations and molecular pathways is necessary in order to provide more insightful diagnostic tools and therapeutic agents.

#### 2.3.2. DNA Methylation in RCM

There have been no studies to date regarding changes in global DNA methylation in RCM. However, a recent study by Glezeva et al. performed targeted DNA methylation profiling of human cardiac tissue in order to determine alterations in DNA methylation and the associated dysregulation of gene expression amongst various heart failure models from cardiac tissue of different patients. As this study included patient samples from other types of CMPs, the loci revealed require further investigation in the context of CMPs. Such loci could be potentially altered in RCM and could be critical for determining disease-associated gene expression changes as a result of aberrant methylation patterns [84].

#### 2.3.3. Histone Modifications in RCM

Very few studies have outlined a specific role of histone modifications in RCM. Zhao et al. revealed that phosphodiesterase (PDE) 4d is downregulated in the heart of mice with restrictive cardiomyopathy, an effect associated with enhanced acetylation of histone 3 lysine 4 and lysine 9 and decreased trimethylation of histone 3 lysine 4, in the promoter region of PDE4d. Moreover, an increase in binding of the histone transmethylase SMYD1 and histone deacetylase HDAC1 was observed in the promoter region of mutated cTnI. The overall findings of this study indicated that the decrease in PDE4d in RCM mice as a result of cTnI mutations could be regulated by a balance between histone acetylation and methylation [56]. cTnI is a subunit of the thin filament involved in regulation of heart contraction; mutations in cTnI have been shown to be most prevalently associated with RCM. Mutations in cTnI repress PDE4d expression in cardiomyocytes via an HDAC1-associated mechanism, potentially regulating gene expression associated with RCM pathogenesis [57] (Table 2). These studies provide insight into a potential epigenetic mechanism controlling RCM, and further analyses are necessary to fully understand how the epigenome is altered in pathological RCM.

#### 2.3.4. Chromatin Remodeling in RCM

There are currently no studies describing chromatin remodeling mechanisms in RCM; however, of note is a potential mechanism involving nuclear intermediate filament networks formed by A- and B-type Lamins, which are major components of the nucleoskeleton and have been implicated in various pathophysiologies. Biochemical studies implicating Lamin–F-actin binding disruption as a result of mutations to Lamin A indicated that concentration of free actin in the nucleus could impact transcription, nuclear export, chromatin remodeling, chromatin movement, and nuclear assembly [85]. This study could be an important stepping stone in the regulation of chromatin remodeling mechanisms in RCM as a result of mutations in structural genes; however, further study is still necessary to elucidate these pathways. 

#### 2.3.5. Noncoding RNAs in RCM

Only one study to date has implicated noncoding RNAs in the pathophysiology of RCM. This study aimed to investigate transcriptomic changes occurring in cardiac tissues of patients with HF, in order to further elucidate the molecular mechanisms involved. Interestingly, this study used only two dilated cardiomyopathy (DCM) and two restrictive cardiomyopathy (RCM) patient samples to perform transcriptomic analysis, identifying DCM- and RCM-specific expression signatures for protein-coding genes, as well as for lncRNAs. This study was the first to reveal 27 lncRNA/mRNA pairs that were significantly altered in HF patients compared to control samples [86], providing essential data for further studies on noncoding RNAs as potential regulators of RCM.

### 2.4. Arrhythmogenic Cardiomyopathy

#### 2.4.1. Molecular Mechanisms in ACM: An Overview

Over the last few years, several molecular pathways have been elucidated in the pathophysiology of ACM, including Wnt/β-catenin and Hippo signaling. These pathways have been shown to be perturbed in response to the causal mutations of ACM, and aberrant activity of these mechanisms has been shown to result in myocyte death, fibro-adipogenesis, and gap junction and ion channel remodeling, which lead to myocardium wall thinning, progressive heart failure, current conduction deficiencies, and arrhythmias [87]. In patients with ACM, molecular remodeling of the intercalated discs leads to pathogenic activation of the Hippo pathway, inactivation of its downstream effector YAP, and enhanced adipogenesis [88]. The Hippo pathway is involved in growth, differentiation, and proliferation of a myriad of cell types; activation of this pathway in ACM patients results in suppression of gene expression of its downstream targets, as well as suppression of the canonical Wnt signaling pathway through β catenin. Such events lead to aberrant changes in cardiomyocyte homeostasis, resulting in the ACM phenotype [80,89]. The crosstalk between the Hippo/YAP pathway and the canonical Wnt/β-catenin pathway contributes to the pathogenesis of ACM; YAP (Yes-associated protein) is known to interact with β-catenin to prevent its nuclear localization, thereby inhibiting Wnt signaling. Since the Hippo pathway is primarily controlled through cell adhesion and polarity, cell adhesion at the intercalated disc in cardiomyocytes is essential for mechanical continuity and electrical propagation throughout the myocardium. Moreover, this cell adhesion is necessary for scaffolding of β-catenin [88,90]. Suppression of the Wnt signaling pathway leads to adipogenesis as a result of abnormal distribution of intercalated disc proteins and, furthermore, results in fibrogenesis and apoptosis. The interaction between YAP and β-catenin in the nucleus is known to promote Wnt-related gene expression. In a murine model of PKP2 knockdown, as well as in ACM patients, aberrant activation of the Hippo kinase cascade resulting in phosphorylation and cytoplasmic retention of YAP was shown, leading to suppression of canonical Wnt signaling. This results in enhanced myocyte death and fibro-adipogenesis, contributing to the pathogenesis of ACM [91]. 

#### 2.4.2. DNA Methylation in ACM

Changes in global DNA methylation have not been reported in ACM to date; however, this will be a key direction for future studies, especially in the context of Hippo and Wnt/β-catenin pathways. A recent study showed that downregulation of YAP in mouse ESCs caused a genome-wide alteration of the DNA methylation remodeling that takes place during the early steps of differentiation [92]. Moreover, the Wnt/β-catenin and TGF-β signaling pathways have been implicated in the modulation of methylation patterns in epithelial–mesenchymal transition (EMT) in esophageal squamous cell carcinoma [93]. Indications that these molecular pathways have been shown to be involved in genome-wide methylation changes in other cellular and pathological contexts is a very promising direction for future studies in the pathophysiology of ACM and in unraveling the specific pathways involved in this disease. 

#### 2.4.3. Histone Modifications in ACM

Histone modifications as epigenetic regulators of ACM have not been described in detail. Studies have shown that electrophysiological differences between the right and left ventricles can potentially contribute to ACM. For example, Brugada syndrome is an inherited arrhythmogenic disorder causing sudden death, which has been associated with mutations in 18 different genes. Loss-of-function mutations in the *CACNA1C* and *CACNB2* genes account for up to 12% of Brugada syndrome cases [94]. Moreover, studies have shown that inherited mutations of *SCN5A* (Na^+^ channel) and *HEY2* (Notch signaling pathway) can result in ventricular arrhythmias and sudden cardiac death [58]. Several studies have also outlined conduction defects in the failing heart that are associated with dysregulation of repolarizing K^+^ currents, depolarizing Ca2^+^ and Na^+^ currents, Ca2^+^ transporters (*NCX, SERCA2a*), and downregulation of the cardiac gap junction protein Connexin 43 [59]. The KCNIP2 (potassium channel interacting protein 2), which interacts with the subfamily of voltage gated potassium channel (Kv4), regulating cardiomyocyte currents, has been shown to be regulated by Notch signaling in mouse models of heart failure, whereby downregulation of Kcnip2 is correlated with a loss of H3K4me3 associated with dynamic RBP-J binding to the *Kcnip2* promoter [60]. A different study comparing left- and right-ventricular changes in histone modifications at the level of atrial natriuretic peptide (ANP) and brain natriuretic peptide (BNP) found that there were higher levels of H3K4me2 and H3K9ac (activating modifications) with concomitantly higher expression levels of ANP and BNP in the LV compared to RV. On the other hand, it was observed that there is an upregulation of both ANP and BNP in the failing LV, with a reduction in H3K9me2 and H3K9me3 (repressive modifications) [58]. These aberrantly regulated genes could be potential targets for further studies of epigenetic regulation in ACM (Table 2).

A very important study as a stepping stone for correlating epigenetic modifications to ACM pathology used high-throughput RNA-sequencing analysis, identifying more than 5000 differentially expressed genes, correlated with suppression of the Hippo and canonical Wnt pathways. These genes were tested in right- and left-ventricular tissues from five independent ACM patients. Downregulation of Wnt and Hippo pathways was observed; however, levels of the histone acetyltransferase p300, which is known to suppress the Hippo and Wnt pathways, was increased, providing a novel mechanism of epigenetic control in ACM [61]. 

In summary, these results indicate the involvement of changes to histone modifications and the epigenetic landscape of the cardiomyocyte in response to pathological stimuli. It will be of great interest to study global changes in histone modifications using chromatin immunoprecipitation followed by high-throughput sequencing in patient samples with ACM, in order to fully understand how mutations in desmosomal proteins, via downstream regulation of Hippo/Wnt/β-catenin pathways, can alter the epigenome, resulting in the pathophysiology of ACM.

#### 2.4.4. Chromatin Remodeling in ACM

Mechanisms of chromatin remodeling still remain elusive in the context of ACM; however, an interesting direction to take for future studies is the use of the genome-wide chromatin conformation capture (Hi–C) assay, which was recently applied in a pressure-overload murine model of cardiac hypertrophy. This study demonstrated alterations in chromatin compartmentalization and looping, indicating correlations between chromatin remodeling coupled with decreased enhancer interactions in heart failure [95]. Interestingly, the Hippo signaling pathway, specifically its downstream components YAP/TAZ/TEAD, has been shown to be associated to enhancer elements at the genome-wide level in cancer cells [96]. Although changes in chromatin conformation and remodeling have not been studied in ACM, these two studies could be very indicatory of a promising research direction in divulging a role for epigenetics in the pathophysiology of this disease.

#### 2.4.5. Noncoding RNAs in ACM

Causative mutations of ACM have been shown to affect miRNA profiling in cardiac tissue. A recent study used two transgenic murine models carrying mutations found in ACM patients. Not only did these mice present with decreased Wnt/β-catenin signaling, but genome-wide RNA-sequencing showed upregulation of miR-217-5p and miR-708-5p and a reduction in miR-499-5p [81]. Therefore, these novel miRNAs will be important in future studies of epigenetic regulation in ACM patients. Another study screened for differentially expressed miRNAs in order to functionally characterize the effects of these miRNAs in cardiac mesenchymal progenitor cells in order to corroborate the ACM phenotype. This study found that PKP2 deficiency leads to suppression of the E2F1 pathway, resulting in hypermethylation of the miR-184 promoter. This led to a decrease in miR-184 levels contributing to the onset of adipogenesis [80], providing yet another potential epigenetic target for future studies. Moreover, another study screened 750 miRNAs and found that 59 of these were significantly modulated in plakophilin 2-deficient HL-1 cells. This study also found mir-184 to be the most robustly regulated in this context [80] (Table 3). Using miRNAs as a potential diagnostic tool for various pathophysiologies has gained much interest over the last decade. One pivotal study in this regard found a correlation between ACM and low plasma levels of miR-320a; this miRNA appeared to have implications in the pathogenesis of ACM, was shown to be increased during adipogenic differentiation of human mesenchymal bone marrow cells, and was found to regulate the Wnt pathway in human colon cancer cells [97]. 

Lastly, although lncRNAs are emerging as promising epigenetic biomolecules in disease onset and for therapeutic targets, there has not been a comprehensive genome-wide analysis of ACM patient cardiac tissue. This will be key to identifying novel biomolecular targets in ACM, specifically in the context of perturbed Hippo/Wnt/β-catenin signaling.

## 3. Conclusions and Future Perspectives

Cardiomyopathies are an important cause of death worldwide, providing a great burden to healthcare systems. Over the years, several animal and cellular models mimicking the heterogeneous pathophysiologies of CMPs have been developed, contributing to our knowledge of the genetic mechanisms underlying these diseases. Despite a significant improvement of the knowledge of the causal mutations contributing to the various cardiomyopathies, very little is known about a potential role of molecular and epigenetic mechanisms, which will be imperative for development of more comprehensive diagnostic tools and potential novel therapeutics.

In this context, high-throughput omic approaches are necessary for determining global changes to the transcriptome in the four types of cardiomyopathies, in order to compare and contrast global changes in gene expression as a result of the causative mutations of these CMPs. Moreover, transcriptomic analysis of the noncoding genome will be paramount in deciphering the role of ncRNAs as biomolecular regulators of aberrant gene expression patterns, also in addition to their use as diagnostic tools. Genome-wide epigenomic studies are also necessary in order to understand global changes in chromatin structure (ATAC sequencing, HiChiP sequencing), changes in histone modification landscapes (ChIP sequencing), and global methylation changes (MeDIP sequencing). Moreover, such analyses will reveal whether there are switches between the active or repressed chromatin state under pathological conditions, as well as global changes to the epigenetic landscape. Combining these omics approaches will also reveal key epigenetic enzymes that are modulated in CMPs. Another important direction in the study of epigenetics, which has only become popular in the last several years, is that of RNA methylation, which has also been implicated in cardiovascular disease [98]. Determining global changes in methylation at the RNA level will provide an additional level of understanding underlying genetic and epigenetic regulation of CMPs. Combination of these omics approaches in studying the four main cardiomyopathies will provide invaluable information for future therapeutic strategies, including epigenetic drug therapy, which has already become a promising avenue for various pathologies. 

In contrast to other cardiac disorders, including heart failure or myocardial infarction, the use of cardiac biomarkers has not received great attention for the diagnosis or prognosis of genetic cardiomyopathies [99]. So far, the diagnosis and the management of genetic cardiomyopathies mainly rely on genetic testing, clinical symptoms, ECG measurements, and cardiac imaging, to detect functional, structural, and morphological alterations [99]. Cardiac-specific plasma protein biomarkers such as BNP, NT-proBNP, and cTns have been questioned regarding their effective value in the diagnosis of CMPs, since they may not be sufficient for early detection or for a better risk stratification of these genetic disorders. Thus, additional biomarkers are currently needed to better guide the intensity of imaging surveillance, refine current risk stratification criteria, and track disease progression [99]. The use of circulating miRNAs as diagnostic tools has been increasing steadily over the last few years [100]; combining genome-wide RNA sequencing in order to standardize miRNAs specific to each CMP will be an important step in overcoming some of the limitations of current diagnostic tools (Table 3).

Lastly, very recent work unveiled a novel implication of cardiac-specific extracellular vesicles (EVs) with prognostic potential in aortic stenosis patients. “EVs” can be collectively described as membranous structures with lipid bilayers that are produced by most cells, ranging in size from 50 to 200 nm [101]. EVs are necessary for intercellular communication and, along with proteins, peptides, lipids, and noncoding RNAs, comprise the cellular secretome [102]. For this reason, EVs may represent an important biomedical tool, as their molecular content is a snapshot of the “releasing” cell type and its physiological state, and because they can be easily detected in body fluids (e.g., blood and urine) [103]. Although their potential as a prognostic tool is extremely promising, the limited knowledge of EV synthesis, cargo loading, and uptake currently hampers their effective translation into the clinic. Despite these challenges, EVs, along with ncRNAs, could prove to be strong diagnostic tools for cardiomyopathies, indicating that epigenetic mechanisms could be paramount for the development of gene-specific therapeutic strategies.

## Figures and Tables

**Table 1:** Genetic mutations in CMPs.

Gene	Protein	Disease	References
*ACTC1*	Actin alpha cardiac muscle 1	DCM	[8]
*TTN*	Titin	DCM	[8]
*MYH6*	Myosin heavy chain 6	DCM	[8]
*TNNT2*	Troponin T	DCM	[8]
*LMNA*	Prelamin A/C	DCM	[8]
*PLNB*	Cardiac phospolamban	DCM	[8]
*MYH7*	Myosin heavy chain 7	HCM	[11,12]
*MYBPC*	myosin-binding protein C	HCM	[11,12]
*TNNI3, TNNT2*	Cardiac troponin I and T	HCM	[11,12]
*TPM1*	tropomyosin α-1 chain	HCM	[11,12]
*MYL3*	Myosin light chain 3	HCM	[11,12]
*TNNT2*	Cardiac troponin T	RCM	[18]
*TNNI3*	Cardiac troponin I	RCM	[18]
*ACTC1*	Actin alpha cardiac muscle 1	RCM	[18]
*MYH7*	Myosin heavy chain 7	RCM	[18]
*JUP*	Plakoglobin	ACM	[22]
*DSP*	Desmoplakin	ACM	[22]
*PKP2*	Plakophilin-2	ACM	[22]
*DSG2*	Desmoglein-2	ACM	[22]
*DSC2*	Desmocollin-2	ACM	[22]
*PLNB*	Cardiac phospolamban	ACM	[23]
*RYR2*	Cardiac ryanodine receptor	ACM	[23]
*TGF-b3*	Transforming growth factor b 3	ACM	[23]
*TMEM43*	Transmembrane protein-43	ACM	[23]
*SCN5A*	Cardiac sodium channel	ACM	[23]

**Table 2:** Known epigenetic enzymes and histone modifications in CMPs.

Disease	Modification	Enzymes	References
DCM	H3K4me3, H3K9me2, H3K9me3, H3K79me3	EP300, G9A, HDAC1, HDAC2, DOT1L	[47,48,49,50,52]
HCM	H3K9me2	G9A, HDACs, EP300	[54,55]
RCM	H3K4Ac, H3K9Ac, H3K4me3	SMYD1, HDAC1	[56,57]
ACM	H3K4me3, H3K4me2, H3K9Ac, H3K9me2, H3K9me3	EP300	[58,59,60,61]

**Table 3:** Altered miRNAs in CMPs.

miRNAs	Disease	Up or Down Regulated	Cardiac/Circulating	References
miR-17-5p, miR-28, miR-106	DCM	Down	Cardiac	[63]
miR-208	DCM	Up	Cardiac	[64]
miR-1, miR-19a/b	DCM	Down	Cardiac	[65]
miR-21, miR-26, miR-29, miR-30 and miR-133a	DCM	Up	Circulating	[66]
miR-3193, miR-3671	HCM	Up	Cardiac	[74]
miR-487a, miR-654, miR-30d, miR-154	HCM	Down	Cardiac	[78]
1-3p, mir-19b, mir-21, mir-29a, mir-155, mir-221	HCM	Up	Circulating	[79]
miR-184, miR-499-5p	ACM	Down	Cardiac	[80,81]
miR-217-5p, miR-708-5p	ACM	Up	Cardiac	[81]
miR-320a	ACM	Down	Circulating	[81]
